# Association of Early-Stage Breast Cancer and Subsequent Chemotherapy With Risk of Atrial Fibrillation

**DOI:** 10.1001/jamanetworkopen.2019.11838

**Published:** 2019-09-20

**Authors:** Husam Abdel-Qadir, Paaladinesh Thavendiranathan, Kinwah Fung, Eitan Amir, Peter C. Austin, Geoffrey S. Anderson, Douglas S. Lee

**Affiliations:** 1 Department of Medicine, Women’s College Hospital, Toronto, Ontario, Canada; 2 ICES, Toronto, Ontario, Canada; 3 Division of Cardiology and Peter Munk Cardiac Centre, University Health Network, Toronto, Ontario, Canada; 4 Institute of Health Policy, Management, and Evaluation, University of Toronto, Toronto, Ontario, Canada; 5 Division of Medical Oncology, Princess Margaret Cancer Centre, Toronto, Ontario, Canada

## Abstract

**Importance:**

Several types of cancer have been linked to a higher risk of developing atrial fibrillation (AF). Fewer data exist regarding early-stage breast cancer (EBC), for which cardio-oncology concerns are more pertinent.

**Objective:**

To investigate the association of EBC and subsequent chemotherapy with the risk of developing AF.

**Design, Setting, and Participants:**

This was a population-based, retrospective, matched cohort study conducted in Toronto, Ontario, Canada, of 68 113 women diagnosed with EBC between April 2007 and December 2016 who were matched 1:3 to a cancer-free control group based on birth year and receipt of breast imaging. Prevalence of AF before the index date (date of EBC diagnosis) was compared between the cohorts using the McNemar test. Cumulative incidence function curves were used to describe the AF incidence. To study preexisting AF, participants were matched before exclusion for prior AF. For the remaining analyses, we excluded women with prior AF before matching. An analysis was conducted beginning 1 year after the index date (ie, excluding AF diagnoses in year 1), which we stratified by chemotherapy exposure. Multivariable cause-specific regression was used to determine the hazard ratio (HR) associated with EBC relative to the controls and the association of chemotherapy with AF in patients with EBC.

**Exposures:**

Breast cancer and chemotherapy.

**Main Outcomes and Measures:**

Incidence of AF.

**Results:**

A total of 68 113 women with EBC and 204 330 cancer-free controls were included in the study; both groups had a mean (SD) age of 60 (13) years. Of the women with EBC, 44.3% were diagnosed as having stage I breast cancer; 38.7%, stage II; and 13.4%, stage III; cancer stage information was missing for 3.6% of the patients. No difference was observed in preexisting AF prevalence (5.3% in the EBC cohort vs 5.2% in controls; *P* = .21). At 10 years after the index date, the AF incidence was 7.4% (95% CI, 7.1%-7.7%) for women with EBC and 6.8% (95% CI, 6.7%-7.0%) for the controls (*P* < .001). The adjusted cause-specific HR was significantly elevated at year 1 (HR, 2.16; 95% CI, 1.94-2.41) and after year 5 (HR, 1.20; 95% CI, 1.11-1.30) but not during years 2 through 5. Analyses beginning 1 year after diagnosis showed attenuated differences that remained statistically significant: the cumulative incidence of AF at 9-year follow-up was 7.0% (95% CI, 6.7%-7.3%) for patients with EBC and 6.5% (95% CI, 6.3%-6.7%) for the cancer-free controls. The rate of AF was higher in women who received chemotherapy (adjusted HR, 1.23; 95% CI, 1.13-1.35) but was not associated with exposure to anthracyclines or trastuzumab.

**Conclusions and Relevance:**

This study’s findings suggest that patients with EBC may not have a higher prevalence of AF before cancer diagnosis. A higher rate of AF was observed in the first year and after 5 years following the EBC diagnosis. The rate of AF was higher in patients who received chemotherapy but appeared to not be associated with specific cardiotoxic agents. These findings suggest that the early and late periods of increased AF risk in EBC survivors warrant focused research to better understand the underlying causes and subsequent implications.

## Key Points

**Question:**

Are early-stage breast cancer and subsequent chemotherapy associated with a higher risk of atrial fibrillation?

**Findings:**

In a cohort study of 68 113 women diagnosed with early-stage breast cancer and 204 330 age-matched cancer-free controls, women with early-stage breast cancer developed atrial fibrillation at a significantly higher rate than that in the cancer-free controls in the first year and after 5 years following their diagnosis but not in the intervening years. The absolute incidence at 10 years was minimally but significantly higher in women with early-stage breast cancer than in the cancer-free controls (7.4% vs 6.8%); chemotherapy exposure was associated with increased risk of atrial fibrillation; however, an increased risk of atrial fibrillation was not observed with anthracyclines or trastuzumab relative to other types of chemotherapy.

**Meaning:**

This study’s findings suggest that women with early-stage breast cancer may experience a marginal but significant increase in the risk of developing atrial fibrillation compared with age-matched women without cancer.

## Teaser

This matched cohort study investigates whether early-stage breast cancer and subsequent chemotherapy are associated with the risk of developing atrial fibrillation among women in Ontario, Canada, who were diagnosed with early-stage breast cancer compared with women without breast cancer.

## Introduction

The growing interest in cardio-oncology (the field of cardiovascular disease in patients with cancer)^1,2,3^ has been accompanied by concerns about an increased risk of developing atrial fibrillation (AF) following cancer diagnosis,^4,5,6,7,8,9,10,11,12,13,14,15^ as well as a higher risk of cancer following the recognition of AF.^15,16,17,18,19,20^ In fact, AF has been proposed as a marker of an occult malignant neoplasm.^16,17^ The association between AF and cancer may be caused by shared risk factors between the 2 diseases,^4,5,6,18^ direct effects of cancer,^4,5,11,21,22^ or adverse effects of cancer therapy.^4,5,11,12,23^ Because several questions remain unresolved regarding the association between AF and cancer, this topic has been highlighted as a priority for further research.^4^

Cardiovascular disease is a particularly pertinent clinical concern for women diagnosed with early-stage breast cancer (EBC).^3,24^ Many EBC survivors are older than 65 years and have hypertension, diabetes, or left ventricular dysfunction. Accordingly, a diagnosis of AF would translate to a clinically relevant stroke risk for many EBC survivors. However, there are scarce and conflicting data on the risk of AF in this increasingly prevalent group of cancer survivors.^10,12,15,25^ Generally, the treatment of EBC involves less invasive surgical procedures, and these patients are at a lower bleeding risk, making it a better population in which to investigate whether cancer or its treatments are associated independently with AF.

We designed a population-based, retrospective, matched cohort study to investigate the risk of developing AF among patients diagnosed with EBC. We hypothesized that patients with EBC may have a higher risk of AF than that for the age-matched, cancer-free control group and that the rate of AF may be higher in patients treated with cardiotoxic systemic chemotherapy. In addition, we hypothesized that patients with EBC may not have a higher prevalence of AF preceding their cancer diagnosis.

## Methods

### Data Sources

The Ontario Health Insurance Plan provides the residents of Canada’s most populous province with universal coverage for medically necessary services. This study used several population-based administrative databases (described in eAppendix 1 in the Supplement), which were linked using patients’ encrypted Ontario Health Insurance Plan number and held securely at ICES (formerly the Institute for Clinical Evaluative Sciences). ICES is a prescribed entity for the purposes of section 45(1) of Ontario’s Personal Health Information Protection Act^26,27^; consequently, research ethics board approval is not legally required, and informed patient consent was not needed. We used validated algorithms to identify several medical diagnoses using ICES-based data sources.^28,29,30,31,32,33,34,35^ Incidences of AF were identified using an algorithm that incorporates hospital or emergency department diagnostic codes and physician claims. The algorithm had a sensitivity of 71% and specificity of 99% when validated against electronic medical records from Ontario’s family physicians.^36^ This study followed the Strengthening the Reporting of Observational Studies in Epidemiology (STROBE) reporting guidelines.

### Cohort Creation

We identified all patients diagnosed with breast cancer in Ontario between April 1, 2007, and December 31, 2016. We applied specific exclusion criteria (eFigure 1 in the Supplement) to create a cohort of female patients diagnosed with stages I to III breast cancer and without any history of another cancer, chemotherapy, or radiation exposure. The index date was that of EBC diagnosis. We attempted to match each patient with EBC to 3 women of the same birth year without a history of cancer after applying the same exclusion criteria as those for the EBC cohort. The cancer-free controls were required to have received a breast imaging test in the past year within a similar time interval as the patients with EBC to reduce differences in health system contact. We calculated the time between EBC diagnosis and breast imaging for each patient with EBC and assigned the same lag time after the control’s imaging date to determine her index date.

To assess the differences in AF prevalence before the index date, we matched patients with EBC to the cancer-free controls before excluding women with prior AF. To create the cohort used for all remaining study analyses, we excluded women with prior AF and then matched patients with EBC to their respective controls. The primary outcome was the development of AF. Death from any cause was treated as a competing risk. The date of last follow-up was March 31, 2018.

### Statistical Analysis

The data were analyzed between January 2018 and April 2019. The baseline characteristics of the EBC and control cohorts were summarized using means (with SDs) for continuous variables and counts (with percentages) for discrete variables. The magnitudes of differences between groups were compared using weighted standardized differences because they are not influenced by large sample sizes. The proportion of patients with prior AF in the first pair of matched cohorts was compared using the McNemar test.

The cumulative incidence function^37^ was used to describe the risk of AF over time in the second pair of matched cohorts. Fine-Gray regression with adjustment for clustering within matched sets was used to test for statistically significant differences in AF incidence between patients with EBC and the cancer-free controls.^38^ Next, we developed a multivariable cause-specific hazard regression model, with EBC status as the key risk factor and time to AF as the outcome while accounting for matched sets. Model covariates included age, year of cohort entry, rural residence, ischemic heart disease, heart failure, diabetes, hypertension, peripheral vascular disease, stroke, chronic obstructive pulmonary disease, and chronic kidney disease. We also adjusted for the number of family physician and specialist claims (updated annually) as a time-varying covariate to account for differences in health system contact. The proportional hazards assumption for EBC status was violated; therefore, we calculated the hazard ratio (HR) annually until year 5.

After observing markedly higher AF rates in year 1 among patients with EBC, we generated another set of cumulative incidence function curves beginning 1 year after the index date (ie, excluding AF diagnoses in year 1) for living patients with EBC and their living matched controls to explore whether the AF diagnosed in year 1 was transient. This analysis was stratified based on chemotherapy exposure in the year following EBC diagnosis to study its association with the risk of AF.

We then developed a second cause-specific regression model limited to patients with EBC to understand the association between their characteristics and the rate of AF. This model included the receipt of any chemotherapy as a time-varying covariate. Next, we developed a third cause-specific regression model to study the association between exposure to anthracycline and trastuzumab with the rate of AF. This analysis was restricted to patients with EBC who had documented chemotherapy regimens for EBC within the 365 days following cancer diagnosis to reduce confounding by indication (ie, not receiving chemotherapy because of comorbidity or frailty). The first date of chemotherapy served as the index date in this analysis. Chemotherapy exposure was modeled as a time-varying covariate with 4 potential categories as previously described^39,40^: anthracycline-based chemotherapy without trastuzumab, trastuzumab without anthracyclines, anthracycline-based therapy followed by trastuzumab, and other chemotherapy without anthracyclines or trastuzumab (reference category). Both models analyzing chemotherapy exposure additionally censored women when documented with distant metastases because they may be switched to different chemotherapy regimens. We repeated both analyses in patients with EBC who were 66 years or older to additionally adjust for baseline medication use and time-varying covariates modeling endocrine therapy exposure after the index date. In a post hoc exploratory analysis, we examined the association of AF with cancer recurrence (eAppendix 2 in the Supplement).

Since this study used administrative data sets from a universal health care system encompassing the entire population, we assumed that missing data were negligible unless otherwise stated. Most analyses were conducted using SAS, version 9.4 (SAS Institute Inc). The matched Fine-Gray regression analyses were conducted using the crrc function from the crrSC package for R (R Project for Statistical Computing). All statistical tests were 2-sided. Statistical significance was defined as a 2-tailed *P* < .05.

## Results

A total of 95 539 women diagnosed with breast cancer during the accrual period were considered for cohort creation. As illustrated in eFigure 1 in the Supplement, during cohort creation, we excluded 3793 patients with EBC (5.3%) who had previously documented AF from the 71 906 otherwise eligible women. The first matched sample (which did not exclude women with prior AF) included 217 456 cancer-free women with otherwise similar exclusion criteria. Of these, 11 344 (5.2%) had previously received a diagnosis of AF. No significant difference was observed in the proportion of women with prior AF between patients with EBC and their matched controls (5.3% vs 5.2%, respectively; *P* = .21).

After excluding women with prior AF, we identified 68 113 patients with EBC, who were matched to 204 330 cancer-free women. The baseline characteristics of this matched sample are summarized in Table 1. The mean (SD) age was 60 (13) years for both cohorts. Of the women with EBC, 44.3% were diagnosed as having stage I breast cancer; 38.7%, stage II; and 13.4%, stage III; cancer stage information was missing for 3.6% of the patients. The weighted standardized differences between the 2 cohorts were less than 0.05 for all baseline variables, indicating that the patients with EBC were well matched to their controls on the measured characteristics.

**Table 1.** Baseline Characteristics of Patients With Breast Cancer and Matched Control Patients

Characteristic	No. (%)		Weighted Standardized Difference^a^
	Patients With Breast Cancer (n = 68 113)	Matched Controls (n = 204 330)	
Age, mean (SD), y	60 (13)	60 (13)	0.02
Income quintile			
1	11 775 (17.3)	35 314 (17.3)	<0.01
2	13 280 (19.5)	39 314 (19.2)	0.01
3	13 455 (19.8)	40 642 (19.9)	<0.01
4	14 211 (20.9)	43 185 (21.1)	0.01
5	15 254 (22.4)	45 393 (22.2)	<0.01
Rural residence	8205 (12.0)	22 942 (11.2)	0.03
Hospitalization(s) for MI	442 (0.6)	1261 (0.6)	<0.01
Percutaneous coronary intervention	455 (0.7)	1305 (0.6)	<0.01
Coronary artery bypass surgery	120 (0.2)	339 (0.2)	<0.01
Ischemic heart disease	3750 (5.5)	11 949 (5.8)	0.01
Heart failure	1456 (2.1)	4167 (2.0)	0.01
Heart failure hospitalization	310 (0.5)	907 (0.4)	<0.01
Any prior cardiac disease	5079 (7.5)	15 957 (7.8)	0.01
Diabetes	10 848 (15.9)	31 587 (15.5)	0.01
Hypertension	29 592 (43.4)	87 035 (42.6)	0.02
Peripheral vascular disease	950 (1.4)	2777 (1.4)	<0.01
Stroke	254 (0.4)	1128 (0.6)	0.03
Chronic obstructive pulmonary disease	2644 (3.9)	7451 (3.6)	0.01
Chronic kidney disease	1571 (2.3)	4952 (2.4)	0.01
Chronic dialysis	74 (0.1)	294 (0.1)	0.01
Breast cancer–specific characteristics			
Chemotherapy	36 222 (53.2)	NA	NA
Radiotherapy	48 816 (71.7)	NA	NA
Mastectomy	26 666 (39.1)	NA	NA
Breast cancer stage at diagnosis			
I	30 177 (44.3)	NA	NA
II	26 346 (38.7)	NA	NA
III	9127 (13.4)	NA	NA
Missing stage data	2463 (3.6)	NA	NA
Breast cancer side			
Bilateral disease	631 (0.9)	NA	NA
Left	34 302 (50.4)	NA	NA
Right	33 168 (48.7)	NA	NA
Unknown	12 (<0.01)	NA	NA

Abbreviations: MI, myocardial infarction; NA, not applicable.

^a^ Standardized differences less than 0.1 indicate a minimal magnitude of difference between the groups.

**Figure 1.**
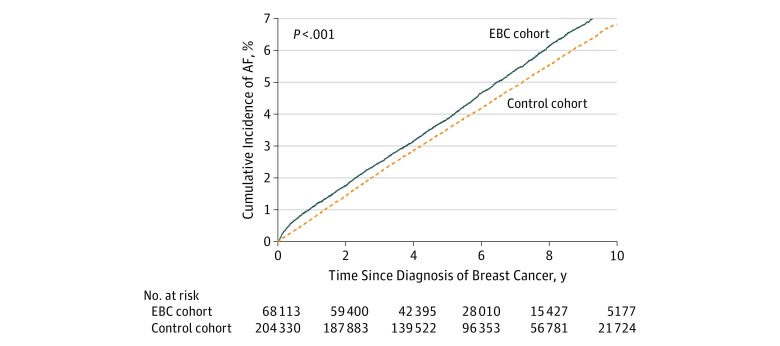
Risk of Atrial Fibrillation (AF) in Early-Stage Breast Cancer (EBC) and Cancer-Free Control Cohorts Cumulative incidence function curves displaying the risk of AF over time among women with EBC and among cancer-free women matched on age and receipt of breast imaging before the index date.

During a mean (SD) follow-up of 5.7 (2.9) years, AF was observed in 3131 patients with EBC (4.6%) and 8793 cancer-free controls (4.3%). Death before AF occurred in 7519 patients with EBC (11.0%) and 10 724 cancer-free controls (5.2%). Figure 1 shows that patients with EBC had a higher cumulative incidence of AF compared with their cancer-free controls despite having higher competing risks from death. At 10 years after diagnosis, the risk of AF was 7.4% (95% CI, 7.1%-7.7%) for patients with EBC and 6.8% (95% CI, 6.7%-7.0%) for the controls (*P* < .001). This translated to approximately 1 extra case for every 189 women after 10 years. The corresponding cumulative incidence of death before AF was 18.5% (95% CI, 18.0%-18.9%) for patients with EBC and 8.9% (95% CI, 8.7%-9.1%) for the cancer-free controls.

**Figure 2.**
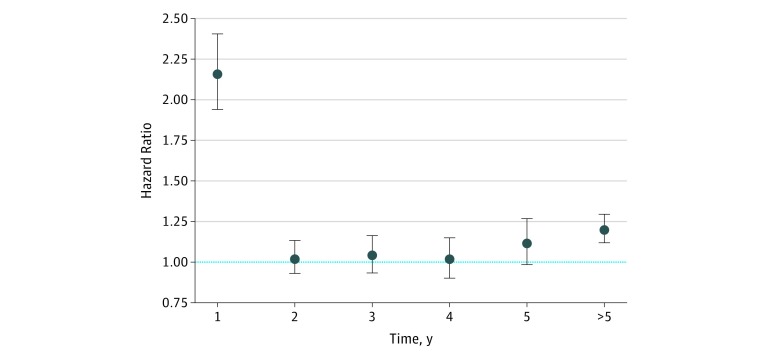
Hazard Ratio of Atrial Fibrillation (AF) After Early-Stage Breast Cancer (EBC) Adjusted cause-specific hazard ratios (HRs) for AF among patients with EBC relative to cancer-free controls. Because the proportional hazards assumption was violated, we present the HRs annually for the first 5 years. The blue horizontal dashed line at 1.00 indicates no difference. Error bars indicate 95% CI.

A greater number of family physician and specialist fee claims was observed for patients with EBC compared with that for the cancer-free controls, although these differences decreased over time (eFigure 2 in the Supplement). Figure 2 illustrates the cause-specific hazard ratios for AF among patients with EBC relative to those in cancer-free controls for years 1 through 5 and thereafter after adjusting for baseline characteristics and physician contact (eFigure 3 in the Supplement provides the unadjusted values, and eTable 1 in the Supplement gives the full-model results). Patients with EBC had a significantly higher rate of AF in the first year (HR, 2.16; 95% CI, 1.94-2.41) relative to that for the controls but no significant differences in years 2 through 5. After year 5, the rate of AF was significantly higher in the EBC cohort (HR, 1.20; 95% CI, 1.11-1.30) relative to that in the cancer-free controls.

**Figure 3.**
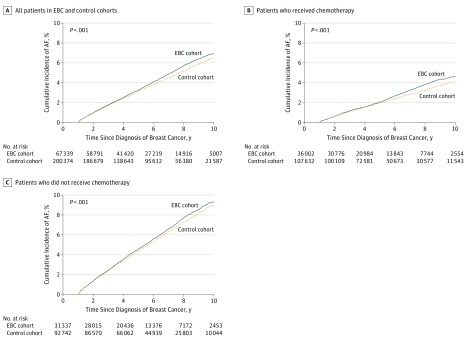
Risk of Atrial Fibrillation (AF) After the First Year Following Breast Cancer Diagnosis Cumulative incidence function curves showing the risk of AF over time in patients with early breast cancer (EBC) and their matched controls beginning at 1 year after the index date.

In the analysis beginning 1 year after breast cancer diagnosis, the cumulative incidence function curves (Figure 3) showed a comparable risk of AF in both cohorts between years 2 through 5. Subsequently, an increased risk of AF was observed in the EBC cohort compared with that in the cancer-free controls. The cumulative incidence at the 9-year follow-up (ie, 10 years after diagnosis) was 7.0% (95% CI, 6.7%-7.3%) for patients with EBC and 6.5% (95% CI, 6.3%-6.7%) for the controls (*P* < .001). After stratifying the EBC cohort by chemotherapy exposure during year 1, the relative risk increase was more prominent in women who received chemotherapy. This was a younger group of patients (mean [SD] age, 56 [12] years); thus, the incidence of AF was lower in these women and their matched controls. The risk of AF in patients with EBC who received chemotherapy was comparable with that of their matched controls in years 2 through 5, after which the curves diverged, indicating a higher risk of AF with long-term follow-up. The cumulative incidence of AF at the 9-year follow-up (ie, 10 years after diagnosis) was 4.7% (95% CI, 4.3%-5.0%) for patients with EBC and 4.2% (95% CI, 4.0%-4.4%) for the controls (*P* < .001). Among the patients with EBC who did not receive chemotherapy in year 1 (mean [SD] age, 65 [13] years), the relative risk of AF was smaller. The cumulative AF incidence at the 9-year follow-up (ie, 10 years after diagnosis) was 9.4% (95% CI, 8.9%-9.9%) for patients with EBC and 9.0% (95% CI, 8.7%-9.3%) for the controls (*P* < .001).

The multivariable cause-specific hazard regression model limited to patients with EBC (full results in eTable 2 in the Supplement) showed that older age, prior cardiovascular diagnoses, hypertension, diabetes, chronic obstructive pulmonary disease, and chronic kidney disease were associated with an increased rate of AF. Patients with stage III disease had a higher AF rate relative to those with stage I disease. Chemotherapy exposure was associated independently with an increased rate of AF, with an adjusted cause-specific HR of 1.23 (95% CI, 1.13-1.35; *P* < .001). This was comparable to the HR associated with diabetes (1.20, 95% CI, 1.10-1.31) and hypertension (1.41; 95% CI, 1.29-1.54). We observed similar patterns when the analysis was limited to patients 66 years or older while also adjusting for endocrine therapy as time-varying exposures and other medications at baseline.

As indicated in eFigure 1 in the Supplement, the chemotherapy-specific analysis included 30 102 women, among whom 755 AF events occurred. Their characteristics are reported in Table 2. In general, women who received anthracycline treatment were younger and healthier than those who received anthracycline-free regimens. eTable 3 in the Supplement summarizes the results of the multivariable cause-specific hazard regression model examining the association of the AF rate with exposure to anthracyclines, trastuzumab, or sequential therapy relative to other chemotherapy without anthracyclines or trastuzumab. In that analysis, anthracycline and trastuzumab exposures were not associated with an increased rate of AF compared with regimens not containing these agents. We observed a similar pattern in the analysis accounting for endocrine therapy and other prescription medications in women 66 years or older. The post hoc sensitivity analysis, in which documentation of metastases was treated as a competing risk, showed attenuated differences that remained statistically significant (eAppendix 2 in the Supplement).

**Table 2.** Baseline Characteristics of Patients With Early-Stage Breast Cancer and Documented Exposure to Specific Chemotherapy Regimens

Characteristic	No. (%)^a^				*P* Value
	Anthracyclines; No Trastuzumab (n = 17 012)	Trastuzumab; No Anthracyclines (n = 2348)	Anthracyclines Plus Trastuzumab (n = 6017)	Other Chemotherapy (n = 4725)	
Age, mean (SD), y	53 (10)	60 (12)	52 (11)	58 (11)	<.001
Income quintile					
1	2773 (16.3)	395 (16.8)	978 (16.3)	705 (14.9)	<.001
2	3214 (18.9)	452 (19.3)	1121 (18.6)	888 (18.8)	
3	3318 (19.5)	490 (20.9)	1196 (19.9)	1015 (21.5)	
4	3772 (22.2)	470 (20.0)	1369 (22.8)	954 (20.2)	
5	3901 (22.9)	540 (23.0)	1336 (22.2)	1155 (24.4)	
Unknown neighborhood income	34 (0.2)	<6 Individuals	17 (0.3)	8 (0.2)	
Rural residence	2063 (12.1)	276 (11.8)	677 (11.3)	528 (11.2)	.34
Hospitalization(s) for MI	31 (0.2)	17 (0.7)	10 (0.2)	37 (0.8)	<.001
Percutaneous coronary intervention	41 (0.2)	22 (0.9)	12 (0.2)	47 (1.0)	<.001
Coronary artery bypass surgery	<6 Individuals	10 (0.4)	<6 Individuals	15 (0.3)	<.001
Ischemic heart disease	410 (2.4)	143 (6.1)	149 (2.5)	263 (5.6)	<.001
Heart failure	75 (0.4)	39 (1.7)	24 (0.4)	98 (2.1)	<.001
Heart failure hospitalization	9 (0.1)	<6 Individuals	<6 Individuals	24 (0.5)	<.001
Any prior cardiac disease	513 (3.0)	184 (7.8)	177 (2.9)	346 (7.3)	<.001
Diabetes	1905 (11.2)	402 (17.1)	609 (10.1)	733 (15.5)	<.001
Hypertension	4974 (29.2)	1004 (42.8)	1688 (28.1)	1873 (39.6)	<.001
Peripheral vascular disease	63 (0.4)	36 (1.5)	23 (0.4)	61 (1.3)	<.001
Stroke	23 (0.1)	6 (0.3)	7 (0.1)	15 (0.3)	.03
COPD	320 (1.9)	81 (3.4)	97 (1.6)	151 (3.2)	<.001
Chronic kidney disease	160 (0.9)	59 (2.5)	51 (0.8)	94 (2.0)	<.001
Breast cancer–specific characteristics					<.001
Radiotherapy	14 775 (86.9)	1735 (73.9)	5084 (84.5)	3807 (80.6)	<.001
Mastectomy	8854 (52.0)	1047 (44.6)	3471 (57.7)	1901 (40.2)	<.001
Breast cancer stage at diagnosis					
I	2427 (14.3)	1074 (45.7)	1141 (19.0)	1585 (33.5)	<.001
II	9613 (56.5)	880 (37.5)	3032 (50.4)	2434 (51.5)	
III	4735 (27.8)	342 (14.6)	1768 (29.4)	623 (13.2)	
Missing stage data	237 (1.4)	52 (2.2)	76 (1.3)	83 (1.8)	
Breast cancer side					
Bilateral disease	66 (0.4)	11 (0.5)	14 (0.2)	14 (0.3)	.21
Left	8594 (50.5)	1195 (50.9)	3152 (52.4)	2384 (50.5)	
Right	8350 (49.1)	1142 (48.6)	2851 (47.4)	2326 (49.2)	

Abbreviations: COPD, chronic obstructive pulmonary disease; MI, myocardial infarction.

^a^ Cells with fewer than 6 individuals are suppressed to reduce the risk of reidentification.

## Discussion

In this matched cohort study, newly diagnosed patients with EBC were subsequently diagnosed with AF at higher rates than those for cancer-free women. However, the absolute increase in incidence was small after accounting for competing risks. Patients with EBC developed AF at twice the rate of development for matched controls in the first year following the cancer diagnosis, with a significant but smaller increase in the AF rate after the fifth year. In contrast, no significant difference was observed in the rate of AF during the intervening period. The relative rate of AF was higher in patients with stage III disease and chemotherapy exposure but was not specifically increased by treatment with cardiotoxic agents. We did not observe a higher prevalence of AF before the cancer diagnosis in patients with EBC.

Numerous reports have suggested a higher risk of cancer following an AF diagnosis.^15,16,17,18,19,20^ Many reports have focused on colorectal cancer with conflicting findings,^8,9,10,13,20^ suggesting that this association may reflect detection bias (eg, malignant neoplasm being unmasked by greater health care contact or bleeding in patients starting anticoagulation therapy after an AF diagnosis). In the present study, we did not observe a higher risk of AF preceding the breast cancer diagnosis relative to that in matched cancer-free controls. In contrast to our findings, Wassertheil-Smoller et al^20^ reported an increased rate of new breast cancer diagnoses during long-term follow-up in participants with baseline AF in the Women’s Health Initiative.

Several studies have also reported a higher AF risk following a cancer diagnosis.^4,5,6,7,8,9,10,11,12,13,14,15^ This increased risk has been hypothesized to result from the inflammatory state associated with cancer^4,5,11,21,22^ or the effects of cancer treatment.^4,5,11,12,23^ The higher risk of AF is more commonly reported in the early period following cancer diagnosis. This may reflect postoperative AF because several studies describe this association in cancers that involve extensive surgery, such as colorectal or lung cancer.^8,9,10,14^ Fewer studies assessed AF risk during longer-term follow-up; these mostly showed smaller magnitudes of association between AF and cancer beyond the first year. An analysis of 15 428 patients from the Reasons for Geographic and Racial Differences in Stroke cohort study^6^ investigated the association of AF with cancer by using multivariable logistic regression after excluding patients with active cancer or cancer that was treated within 2 years of the index date. After adjustment, patients with cancer had an odds ratio of 1.19 (95% CI, 1.02-1.38) for development of AF, which is comparable to the adjusted HR (1.20) we observed in 5-year survivors.

There are fewer data specific to patients with EBC despite this being a clinically relevant patient group frequently treated with cardiotoxic chemotherapy. A study of 53 patients with EBC who received anthracyclines showed prolonged intra-atrial and interatrial electromechanical delay, decreased left atrial active and passive emptying volumes, and decreased atrial emptying fraction.^12^ Another study showed a 2% prevalence of AF in presurgery electrocardiograms for patients with breast cancer vs 0.6% in age-matched controls (including 51% males) admitted for noncancer surgery.^10^ A cohort study from Northern Israel reported an increased AF risk in the 90 days after breast cancer diagnosis, but not thereafter.^15^ D’Souza et al^25^ reported an increased AF rate among Danish patients with EBC from 6 months to 3 years, with an increased rate in the first 6 months only among patients younger than 60 years.

An important gap in these studies is that they did not specifically address chemotherapy exposure (which is more common in younger women), differences in health system contact (which introduce a detection bias), or long-term EBC survivorship. Our data indicated that patients with EBC developed AF at a higher rate than that for the matched controls with recent health system engagement despite adjustment for physician contact. This risk was higher in women exposed to chemotherapy. The comparable AF rates in years 2 through 5 and the lack of a specific association with cardiotoxic chemotherapy suggest that the higher rate in year 1 may have been mediated by temporary factors, such as hemodynamic perturbations, fluid shifts, inflammation, or other treatment-related derangements rather than treatment with cardiotoxic agents. Since the absolute risk is small, this finding does not warrant routine surveillance but rather should prompt consideration of AF in the differential diagnosis for women with compatible symptoms. Nonetheless, the implications of the early increase in AF risk should be explored further because postoperative AF following pulmonary lobectomy for lung cancer was associated with an HR of 3.75 (95% CI, 1.44-9.08) for death among 5-year survivors.^14^

The late increase in AF rates among survivors treated with chemotherapy also warrants more study. Previously, we observed that EBC survivors had a higher risk of stroke without being at higher risk for ischemic heart disease. Despite the small differences in the absolute risk of AF, cancer patients may have higher rates of subsequent arterial thromboembolism.^7^ The etiology of this late increase in risk is unclear. Our sensitivity analysis results suggest that this increased risk may be related to cancer recurrence. Alternatively, it may be a manifestation of survival bias or the worsening cardiometabolic profile and weight gain of EBC survivors.^41,42,43,44,45,46^

### Limitations

This study has limitations. Our data were limited to the Canadian province of Ontario, which may not be reflective of other parts of the world despite its ethnically diverse population. Given our reliance on administrative data, we were unable to adjust for risk factors, such as obesity and sleep apnea. We were also unable to study the association of radiation dose to the heart with the risk of AF. Our AF detection algorithm may have underestimated the absolute risk of AF because it prioritizes specificity over sensitivity. Furthermore, our adjustment for health system contact did not account for visit length and testing intensity, including the number of electrocardiograms performed. Moreover, we cannot rule out residual confounding, which may have caused us to underestimate the risk of AF after cardiotoxic chemotherapy. There was a lower prevalence of cardiovascular disease and risk factors among women exposed to cardiotoxic chemotherapy categories. Thus, our observations regarding the association between chemotherapy exposure and AF risk should be interpreted with caution. Finally, because our methods for identifying recurrent cancer have not been validated, that analysis should be considered exploratory.

## Conclusions

Our study findings suggest that a diagnosis of EBC may be associated with a small increase in the risk of AF compared with that for cancer-free women. The relative rate of AF was found to be higher for women who received chemotherapy in the first year following diagnosis. Patients with EBC who survived 5 years after diagnosis were also found to have a higher relative rate of AF, although this rate was less prominent than that in the first year. Our observations suggest that anthracycline or trastuzumab exposure may not be independently associated with a higher rate of AF compared with other forms of chemotherapy. The early and late periods of increased AF risk in EBC survivors warrant focused research to better understand the underlying causes and subsequent implications.

## Notes

Supplement. **eAppendix 1.** Data Sources Used for Study **eAppendix 2.** Exploratory Analysis Examining Impact of Cancer Recurrence on the Risk of Atrial Fibrillation (AF) After Early-Stage Breast Cancer (EBC) **eFigure 1.** Study Flow Diagram **eFigure 2.** Mean Annual Number of Visits With General Practitioners (GPs) and Specialists per Patient in the Early-Stage Breast Cancer (EBC) Cohort and the Matched Control Cohort **eFigure 3.** Unadjusted Cause-Specific Hazard Ratios (HRs) for AF in EBC Patients Relative to Cancer-Free Controls **eTable 1.** Results From the Multivariable Cause-Specific Hazard Regression Model Assessing the Relationship of Early-Stage Breast Cancer (EBC) Status With Atrial Fibrillation While Adjusting for Baseline Characteristics of EBC Patients and Their Matched Controls **eTable 2.** Results From a Multivariable Cause-Specific Hazard Regression Model Assessing the Relationship of Early-Stage Breast Cancer Patient Characteristics With Time to Atrial Fibrillation **eTable 3.** Results From the Multivariable Cause-Specific Hazard Regression Model Assessing the Relationship of Anthracycline and Trastuzumab Exposure With Atrial Fibrillation After Adjusting for Other Characteristics and Exposures of Early-Stage Breast Cancer Patients With Documented Chemotherapy Regimens
