# Supplementary material for: Association of Early-Stage Breast Cancer and Subsequent Chemotherapy With Risk of Atrial Fibrillation
Source: JAMA Netw Open. 2019 Sep 20;2(9):e1911838. doi: 10.1001/jamanetworkopen.2019.11838 (PMC6755537; doi:10.1001/jamanetworkopen.2019.11838)
Supplement: Supplement. — eAppendix 1. Data Sources Used for Study eAppendix 2. Exploratory Analysis Examining Impact of Cancer Recurrence on the Risk of Atrial Fibrillation (AF) After Early-Stage Breast Cancer (EBC) eFigure 1. Study Flow Diagram eFigure 2. Mean Annual Number of Visits With General Practitioners (GPs) and Specialists per Patient in the Early-Stage Breast Cancer (EBC) Cohort and the Matched Control Cohort eFigure 3. Unadjusted Cause-Specific Hazard Ratios (HRs) for AF in EBC Patients Relative to Cancer-Free Controls eTable 1. Results From the Multivariable Cause-Specific Hazard Regression Model Assessing the Relationship of Early-Stage Breast Cancer (EBC) Status With Atrial Fibrillation While Adjusting for Baseline Characteristics of EBC Patients and Their Matched Controls eTable 2. Results From a Multivariable Cause-Specific Hazard Regression Model Assessing the Relationship of Early-Stage Breast Cancer Patient Characteristics With Time to Atrial Fibrillation eTable 3. Results From the Multivariable Cause-Specific Hazard Regression Model Assessing the Relationship of Anthracycline and Trastuzumab Exposure With Atrial Fibrillation After Adjusting for Other Characteristics and Exposures of Early-Stage Breast Cancer Patients With Documented Chemotherapy Regimens [file jamanetwopen-2-e1911838-s001.pdf]

Supplementary Online Content

Abdel-Qadir H, Thavendiranathan P, Fung K, et al. Association of early-stage breast cancer and subsequent chemotherapy with risk of atrial fibrillation. *JAMA Netw Open.* 2019;2(9):e1911838. doi:10.1001/jamanetworkopen.2019.11838

eAppendix 1. Data Sources Used for Study

eAppendix 2. Exploratory Analysis Examining Impact of Cancer Recurrence on the Risk of Atrial Fibrillation (AF) After Early-Stage Breast Cancer (EBC)

eFigure 1. Study Flow Diagram

eFigure 2. Mean Annual Number of Visits With General Practitioners (GPs) and Specialists per Patient in the Early-Stage Breast Cancer (EBC) Cohort and the Matched Control Cohort

eFigure 3. Unadjusted Cause-Specific Hazard Ratios (HRs) for AF in EBC Patients Relative to Cancer-Free Controls

eTable 1. Results From the Multivariable Cause-Specific Hazard Regression Model Assessing the Relationship of Early-Stage Breast Cancer (EBC) Status With Atrial Fibrillation While Adjusting for Baseline Characteristics of EBC Patients and Their Matched Controls

eTable 2. Results From a Multivariable Cause-Specific Hazard Regression Model Assessing the Relationship of Early-Stage Breast Cancer Patient Characteristics With Time to Atrial Fibrillation

eTable 3. Results From the Multivariable Cause-Specific Hazard Regression Model Assessing the Relationship of Anthracycline and Trastuzumab Exposure With Atrial Fibrillation After Adjusting for Other Characteristics and Exposures of Early-Stage Breast Cancer Patients With Documented Chemotherapy Regimens

This supplementary material has been provided by the authors to give readers additional information about their work.

© 2019 Abdel-Qadir et al. *JAMA Network Open.*

eAppendix 1. Data Sources Used for Study

The Canadian province of Ontario provides its residents with universal coverage for medically necessary services, including hospitalizations and physician services, through the Ontario Health Insurance Plan (OHIP). Coverage is administered through an insurance number unique to each patient, which enables ICES to link their data across multiple administrative databases. These data sets are held securely in a linked, de-identified form and analyzed at ICES. ICES is a prescribed entity for the purposes of s. 45(1) of Ontario's Personal Health Information Protection Act, and as such research ethics board approval was not legally required.

The Ontario Cancer Registry (OCR) is an administrative computerized database which stores records of all Ontario residents who have been diagnosed with cancer (except non-melanoma skin cancer) or who have died of it. The main data elements collected are patient demographics, cancer diagnosis details, and death information. Stage data are available for most women diagnosed with breast cancer after 2006. Cancer is not a legally reportable disease in Ontario. However, the Cancer Act provides a legal mandate for Cancer Care Ontario (CCO) to collect data for the OCR. The Act mandates that the data be kept confidential and that it only be used or disclosed for compiling statistics or carrying out medical or epidemiological research.

The OCR is populated with data from the following four sources:

1. pathology reports with any mention of cancer
2. hospital discharge abstracts and day surgery summaries with cancer diagnostic codes
3. records of patients referred to CCO's eight Regional Cancer Centres or the Princess Margaret Hospital (the specialized institutions treating cancer patients in Ontario).
4. death certificates where cancer is recorded as the underlying cause of death

© 2019 Abdel-Qadir et al. *JAMA Network Open.*

Data from sources 2-4 are coded at the source and transmitted to the OCR in electronic form. In contrast, hard copies of pathology reports are sent to the OCR by hospital and private pathology laboratories. These are then coded and input by OCR staff into the computerized database. The OCR currently uses the ICD-O-3 coding system, which is derived from ICD-10 for use in Oncology coding. For breast cancer, the code is C50.1.

Complementing the OCR, the Cancer Activity Level Reporting (ALR) database contains details of systemic therapy administered at regional cancer centers, whereas the New Drug Funding Program (NDFP) database records data on the use of high-cost agents such as trastuzumab and epirubicin. The latter two databases allow determination of cancer treatments received[1,2]. However, there are no validated methods for detecting cancer recurrence.

The Ontario Registered Persons Database (RPDB) records vital statistics, including dates of death. Data on hospital separations across Canada are stored in the Canadian Institute for Health Information Discharge Abstract Database (CIHI-DAD). The National Ambulatory Care Reporting System (NACRS) collects data on hospital-based and community-based ambulatory care, including emergency department (ED) care. The Same Day Surgery (SDS) database stores data on day surgical procedures. Billing claims for physician services are recorded in the OHIP Claims Database. Algorithms have been validated for the identification of several medical diagnoses using these data sources, including acute myocardial infarction (AMI), ischemic heart disease (IHD), heart failure, hypertension, diabetes, renal dysfunction, and chronic obstructive pulmonary disease (COPD) [3-11].

© 2019 Abdel-Qadir et al. *JAMA Network Open.*

References

1. Thavendiranathan P, Abdel-Qadir H, Fischer HD, et al. Breast Cancer Therapy-Related Cardiac Dysfunction in Adult Women Treated in Routine Clinical Practice: A Population-Based Cohort Study. *Journal of clinical oncology : official journal of the American Society of Clinical Oncology.* 2016;34(19):2239-2246.
2. Thavendiranathan P, Abdel-Qadir H, Fischer HD, et al. Risk-Imaging Mismatch in Cardiac Imaging Practices for Women Receiving Systemic Therapy for Early-Stage Breast Cancer: A Population-Based Cohort Study. *Journal of clinical oncology : official journal of the American Society of Clinical Oncology.* 2018:Jco2018779736.
3. Vermeulen MJ, Tu JV, Schull MJ. ICD-10 adaptations of the Ontario acute myocardial infarction mortality prediction rules performed as well as the original versions. *J Clin Epidemiol.* 2007;60(9):971-974.
4. Tu K, Mitiku T, Lee DS, Guo H, Tu JV. Validation of physician billing and hospitalization data to identify patients with ischemic heart disease using data from the Electronic Medical Record Administrative data Linked Database (EMRALD). *The Canadian journal of cardiology.* 2010;26(7):e225-228.
5. Schultz SE, Rothwell DM, Chen Z, Tu K. Identifying cases of congestive heart failure from administrative data: a validation study using primary care patient records. *Chronic Dis Inj Can.* 2013;33(3):160-166.
6. Lee DS, Donovan L, Austin PC, et al. Comparison of coding of heart failure and comorbidities in administrative and clinical data for use in outcomes research. *Med Care.* 2005;43(2):182-188.
7. Atzema CL, Austin PC, Miller E, Chong AS, Yun L, Dorian P. A population-based description of atrial fibrillation in the emergency department, 2002 to 2010. *Annals of emergency medicine.* 2013;62(6):570-577.e577.
8. Tu K, Campbell NR, Chen ZL, Cauch-Dudek KJ, McAlister FA. Accuracy of administrative databases in identifying patients with hypertension. *Open Med.* 2007;1(1):e18-e26.
9. Hux JE, Ivis F, Flintoft V, Bica A. Diabetes in Ontario: determination of prevalence and incidence using a validated administrative data algorithm. *Diabetes Care.* 2002;25(3):512-516.
10. Fleet JL, Dixon SN, Shariff SZ, et al. Detecting chronic kidney disease in population-based administrative databases using an algorithm of hospital encounter and physician claim codes. *BMC Nephrol.* 2013;14:81.
11. Gershon AS, Warner L, Cascagnette P, Victor JC, To T. Lifetime risk of developing chronic obstructive pulmonary disease: a longitudinal population study. *Lancet.* 2011;378(9795):991-996.

© 2019 Abdel-Qadir et al. *JAMA Network Open.*

eAppendix 2. Exploratory Analysis Examining Impact of Cancer Recurrence on the Risk of Atrial Fibrillation (AF) After Early-Stage Breast Cancer (EBC)

This was a post-hoc exploratory analysis. After observing a higher risk of AF in women with EBC beyond the fifth year following their cancer diagnosis, we wanted to explore whether this was driven by cancer recurrence. However, our data sources do not have validated indicators for cancer recurrence. Accordingly, we looked for documentation of metastases beyond one year following cancer diagnosis as a surrogate for cancer recurrence in the absence of validated methods for its detection. We repeated the CIF analyses of AF incidence while treating documentation of metastases after one year of follow-up as a second competing risk in addition to death. Univariable Fine-Gray regression with adjustment for clustering within matched sets was used to test for statistically significant differences in AF risk.

In this analysis, 5893 women with the EBC cohort (8.7%) had documentation of metastases prior to the occurrence of AF or death. As a result, the differences in AF risk between the two cohorts diminish over follow-up. The cumulative incidence of AF at 5 years with this analytic method was 3.7% (3.5 – 3.8%) in the EBC cohort and 3.5% (3.5-3.6%) in matched controls. At 10 years, the cumulative incidence was 6.8% (6.6 – 7.1%) in the EBC cohort and 6.8% (6.7-7.0%) in matched controls. The absolute differences were smaller than those observed in the primary analysis, but the differences remained statistically significant (p-value <0.001).

© 2019 Abdel-Qadir et al. *JAMA Network Open.*

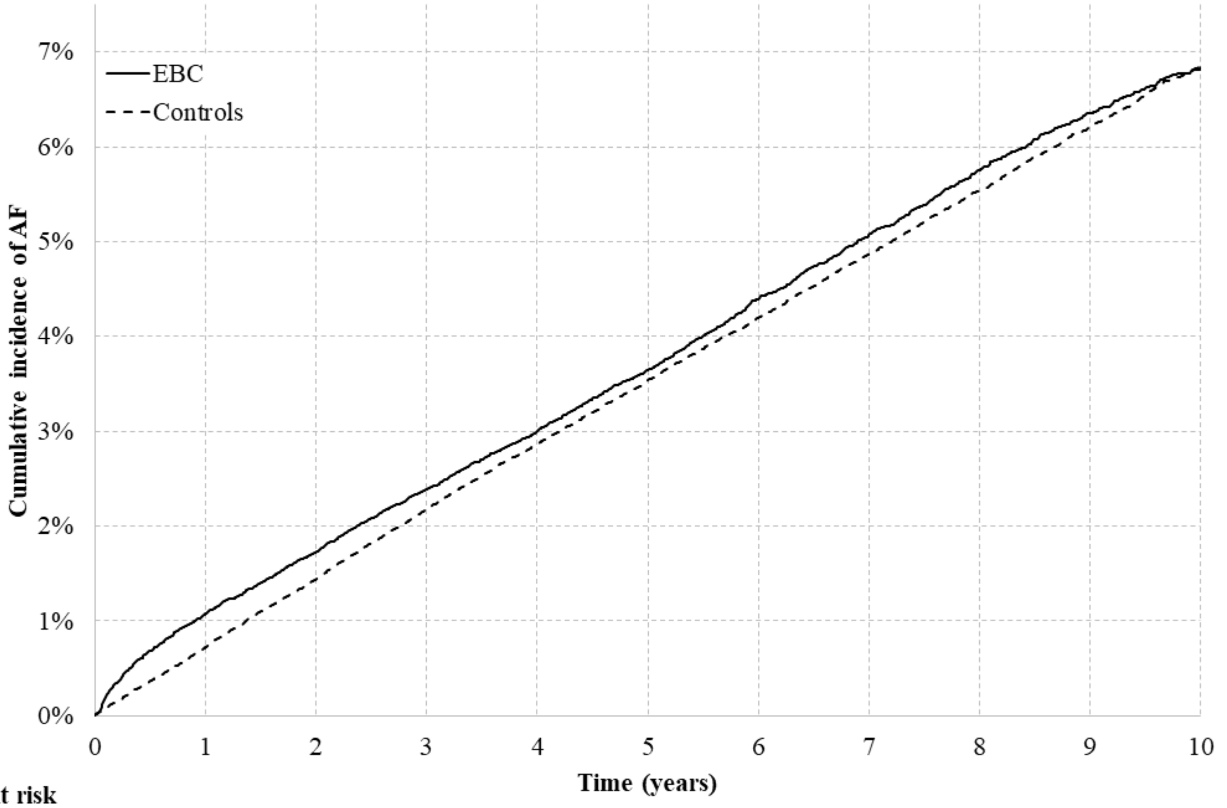

At risk						
EBC	68113	59400	42395	28010	15427	5177
Controls	204330	187883	139522	96353	56781	21724

© 2019 Abdel-Qadir et al. *JAMA Network Open.*

eFigure 1. Study Flow Diagram

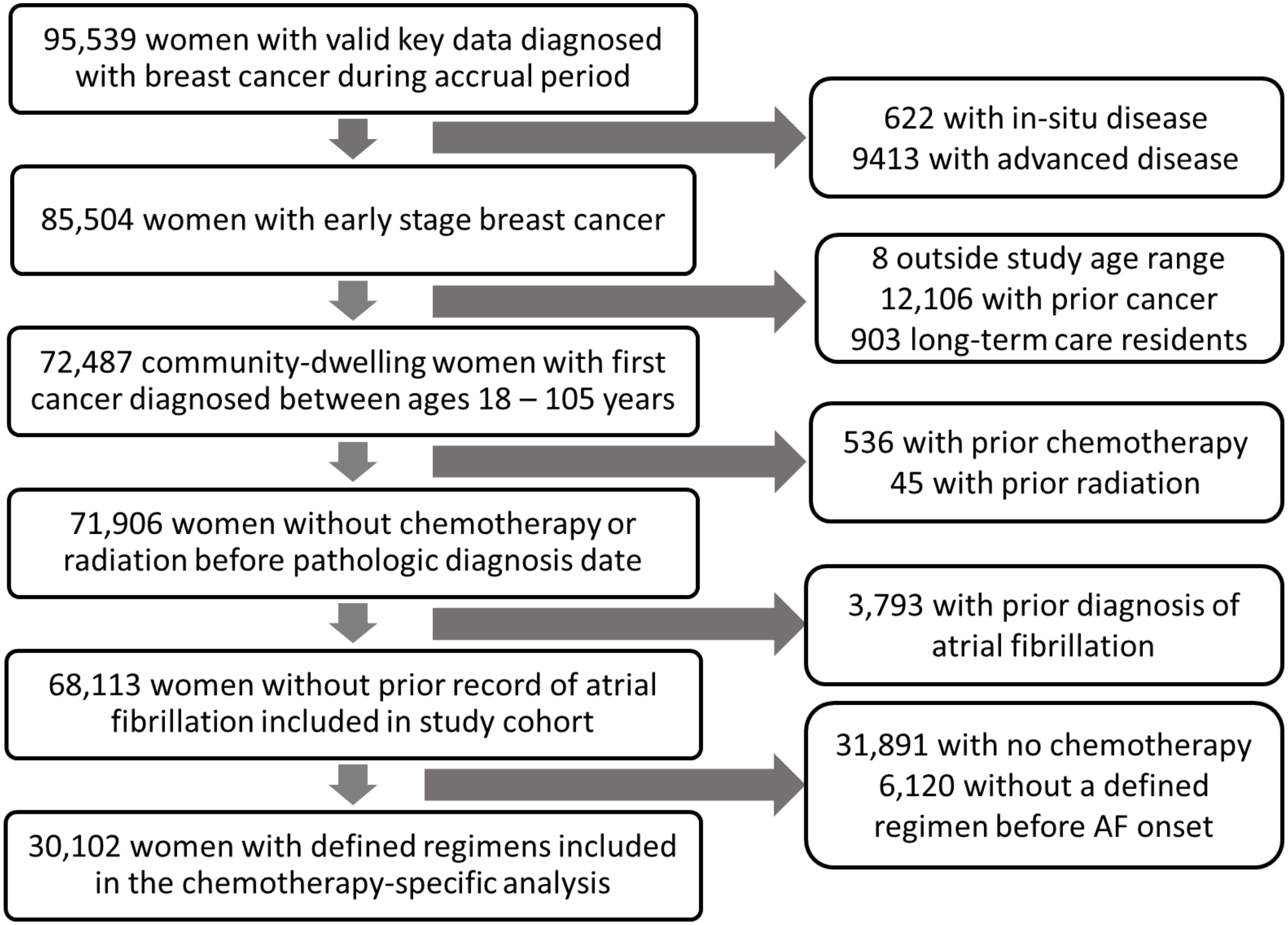

© 2019 Abdel-Qadir et al. *JAMA Network Open.*

eFigure 2. Mean Annual Number of Visits With General Practitioners (GPs) and Specialists per Patient in the Early-Stage Breast Cancer (EBC) Cohort and the Matched Control Cohort

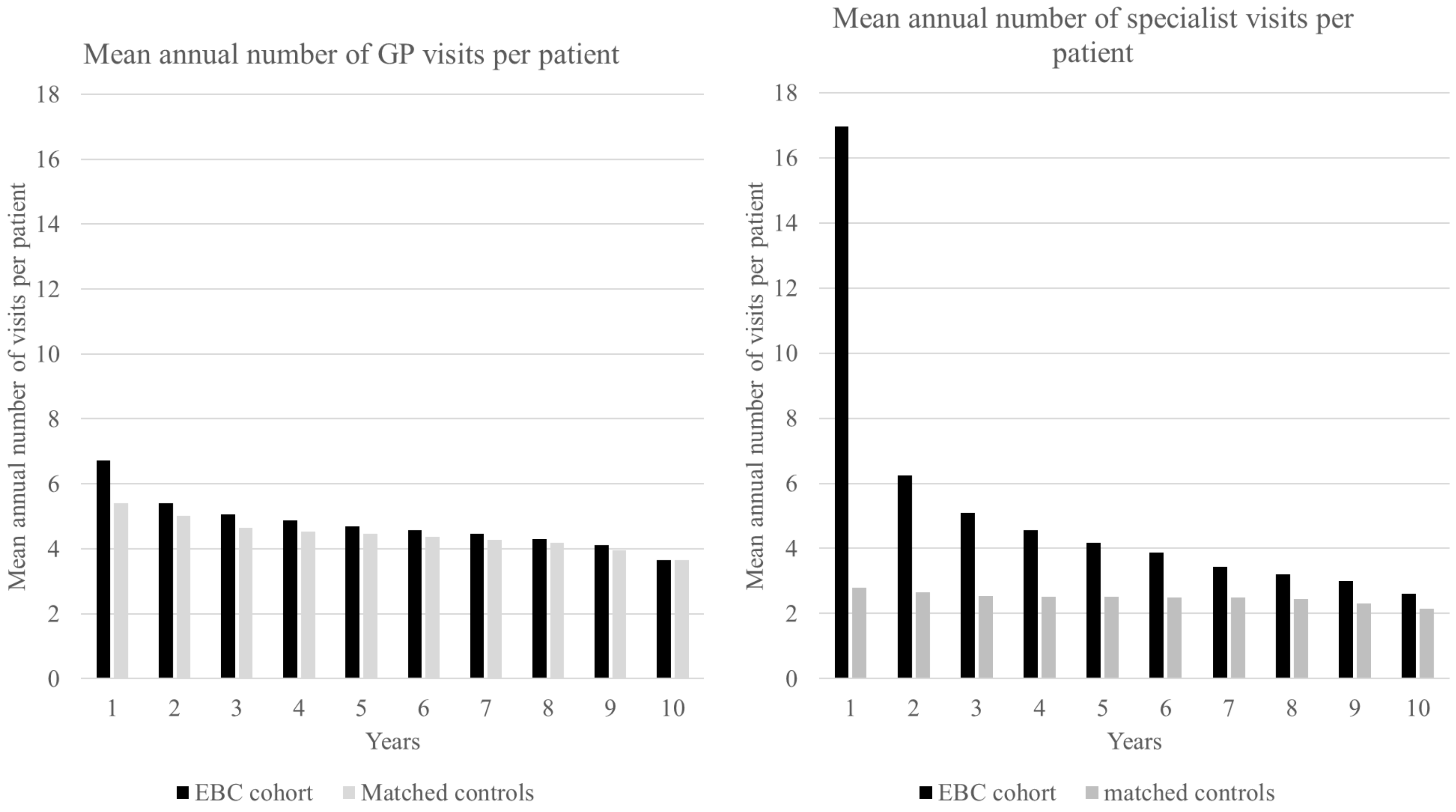

© 2019 Abdel-Qadir et al. *JAMA Network Open.*

eFigure 3. Unadjusted Cause-Specific Hazard Ratios (HRs) for AF in EBC Patients Relative to Cancer-Free Controls. Since the proportional hazards assumption was violated, we present the HR annually for the first 5 years.

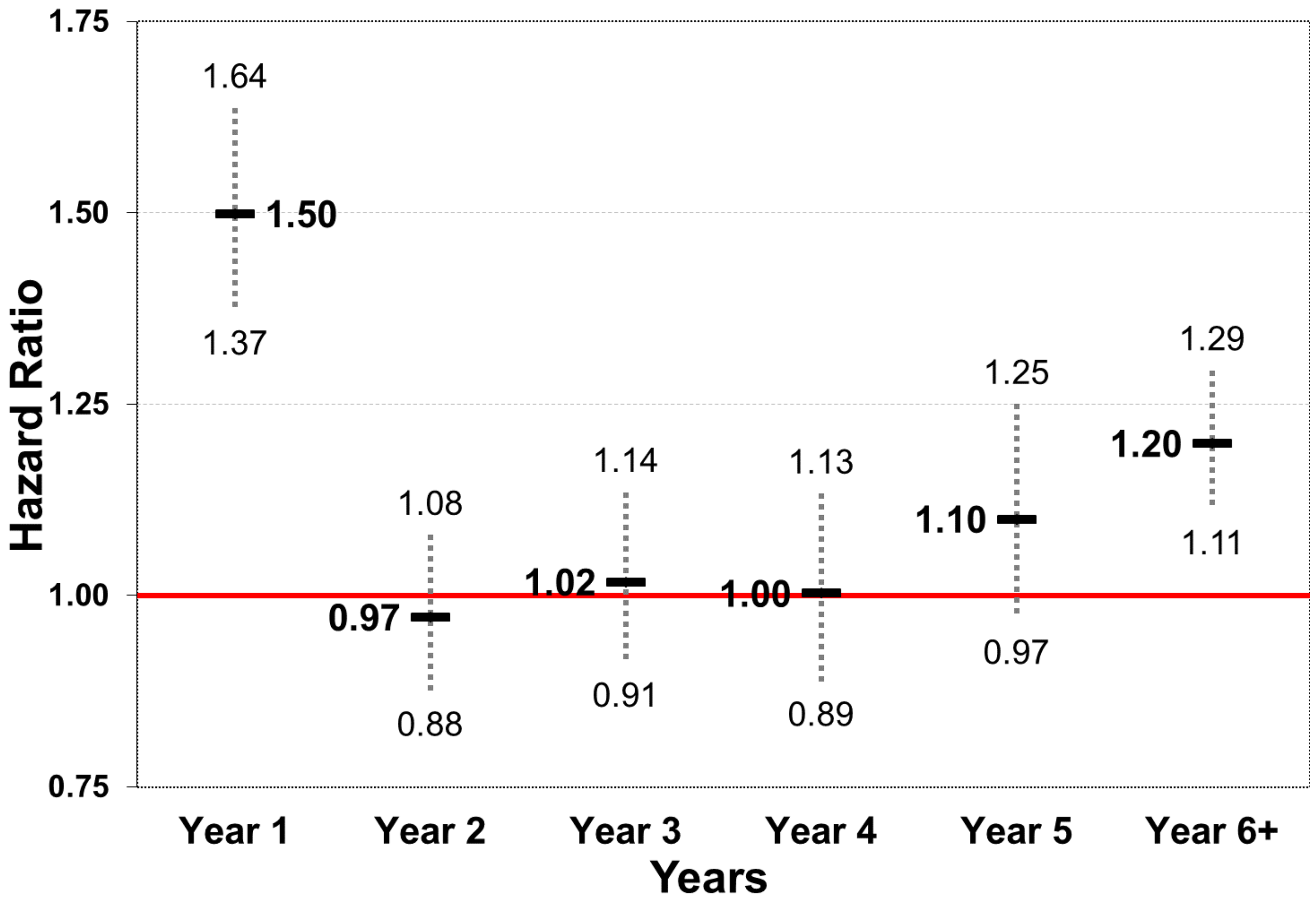

© 2019 Abdel-Qadir et al. *JAMA Network Open.*

eTable 1. Results from the Multivariable Cause-Specific Hazard Regression Model Assessing the Relationship of Early Stage Breast Cancer (EBC) Status With Atrial Fibrillation While Adjusting for Baseline Characteristics of EBC Patients and Their Matched Controls.

	**Hazard Ratio (95% CI)**	**p-value**
**Age (per year)**	1.07 (1.066-1.069)	<0.001
**Year of cohort entry (relative to 2007)**		
**2008**	0.89 (0.83-0.95)	<0.001
**2009**	0.82 (0.77-0.88)	<0.001
**2010**	0.77 (0.72-0.83)	<0.001
**2011**	0.78 (0.73-0.84)	<0.001
**2012**	0.66 (0.61-0.72)	<0.001
**2013**	0.67 (0.62-0.73)	<0.001
**2014**	0.62 (0.57-0.68)	<0.001
**2015**	0.55 (0.49-0.60)	<0.001
**2016**	0.52 (0.46-0.58)	<0.001
**Income quintile (relative to 1 [lowest])**		
**2**	1.00 (0.94-1.06)	0.88
**3**	0.96 (0.90-1.02)	0.14
**4**	0.96 (0.91-1.02)	0.21
**5 (highest)**	0.96 (0.91-1.02)	0.17
**Unknown**	0.80 (0.54-1.17)	0.25
**Urban residence (relative to rural)**	1.06 (1.01-1.12)	0.03
**Ischemic heart disease**	1.53 (1.45-1.62)	<0.001

© 2019 Abdel-Qadir et al. *JAMA Network Open.*

**Heart failure**	1.54 (1.42-1.68)	<0.001
**Diabetes**	1.22 (1.17-1.28)	<0.001
**Hypertension**	1.69 (1.61-1.76)	<0.001
**Peripheral Vascular disease**	1.39 (1.25-1.55)	<0.001
**Stroke**	1.15 (0.96-1.37)	0.14
**Chronic obstructive pulmonary disease**	1.66 (1.54-1.78)	<0.001
**Chronic kidney disease**	1.50 (1.39-1.63)	<0.001
**General practitioner visits in prior year**	0.91 (0.90-0.92)	<0.001
**Specialist visits in prior year**	0.97 (0.96-0.97)	<0.001
**EBC status (relative to cancer-free controls)**		
**Year 1**	2.16 (1.94-2.41)	<0.001
**Year 2**	1.02 (0.92-1.13)	0.73
**Year 3**	1.04 (0.93-1.16)	0.47
**Year 4**	1.02 (0.90-1.15)	0.77
**Year 5**	1.12 (0.98-1.27)	0.09
**After 5 years**	1.20 (1.11-1.30)	<0.001

© 2019 Abdel-Qadir et al. *JAMA Network Open.*

eTable 2. Results From a Multivariable Cause-Specific Hazard Regression Model Assessing the Relationship of Early-Stage Breast Cancer Patient Characteristics With Time to Atrial Fibrillation

	Model in full cohort		Model in patients aged ≥66 years	
**Parameter**	**Hazard ratio (95% CI)**	**p-value**	**Hazard ratio (95% CI)**	**p-value**
**Age (per year)**	1.069 (1.065-1.073)	<0.001	1.06 (1.05-1.06)	<0.001
**Year of cohort entry (relative to 2007)**				
**2008**	0.95 (0.83-1.09)	0.47	1.01 (0.88-1.19)	0.91
**2009**	0.96 (0.84-1.10)	0.60	0.99 (0.84-1.16)	0.86
**2010**	0.93 (0.81-1.07)	0.29	0.98 (0.83-1.16)	0.78
**2011**	0.97 (0.84-1.11)	0.63	1.00 (0.84-1.19)	0.97
**2012**	0.79 (0.67-0.92)	0.004	0.81 (0.66-0.98)	0.03
**2013**	0.79 (0.67-0.93)	0.006	0.83 (0.68-1.00)	0.05
**2014**	0.81 (0.68-0.97)	0.02	0.85 (0.69-1.05)	0.13
**2015**	0.64 (0.52-0.78)	<0.001	0.66 (0.51-0.84)	0.001
**2016**	0.78 (0.63-0.97)	0.03	0.88 (0.68-1.13)	0.31
**Income quintile (relative to 1 [lowest])**				
**2**	0.98 (0.88-1.10)	0.76	1.11 (0.97-1.26)	0.13
**3**	0.86 (0.77-0.97)	0.01	0.96 (0.84-1.11)	0.60
**4**	0.93 (0.83-1.04)	0.23	1.06 (0.93-1.22)	0.39
**5 (highest)**	0.88 (0.79-0.99)	0.03	0.99 (0.86-1.14)	0.86
**Unknown**	1.23 (0.63-2.37)	0.55	1.35 (0.6-3.02)	0.47
**Urban residence (relative to rural)**	0.96 (0.86-1.06)	0.42	0.97 (0.85-1.09)	0.58
**Ischemic heart disease**	1.44 (1.29-1.60)	<0.001	1.28 (1.13-1.45)	<0.001
**Heart failure**	1.45 (1.25-1.68)	<0.001	1.29 (1.08-1.53)	0.004
**Diabetes**	1.20 (1.10-1.31)	<0.001	1.15 (1.04-1.28)	0.007
**Hypertension**	1.41 (1.29-1.54)	<0.001	1.14 (1.00-1.30)	0.05
**Peripheral Vascular disease**	1.32 (1.09-1.60)	0.005	1.12 (0.90-1.39)	0.31
**Stroke**	1.18 (0.81-1.72)	0.39	1.13 (0.74-1.72)	0.56

© 2019 Abdel-Qadir et al. *JAMA Network Open.*

**Chronic obstructive pulmonary disease**	1.51 (1.33-1.71)	<0.001	1.45 (1.26-1.67)	<0.001
**Chronic kidney disease**	1.25 (1.06-1.47)	0.01	1.10 (0.92-1.32)	0.30
**Breast cancer stage (relative to Stage 1)**				
**Stage 2**	1.04 (0.96-1.13)	0.37	1.08 (0.98-1.20)	0.11
**Stage 3**	1.14 (1.01-1.30)	0.04	1.14 (0.98-1.33)	0.10
**Unavailable stage data**	0.89 (0.74-1.07)	0.23	0.96 (0.77-1.18)	0.68
**Left-sided breast cancer**	0.98 (0.92-1.06)	0.66	0.99 (0.91-1.07)	0.74
**Mastectomy (relative to lumpectomy)**	0.99 (0.91-1.08)	0.88	1.00 (0.91-1.11)	0.98
**Radiation exposure (time-varying)**	0.80 (0.73-0.88)	<0.001	0.85 (0.76-0.94)	0.003
**Chemotherapy exposure (time-varying)**	1.23 (1.13-1.35)	<0.001	1.24 (1.11-1.37)	<0.001
**Prescription medications (data only available for patients aged ≥ 66 years)**				
**ACE inhibitor**	-	-	1.03 (0.93-1.15)	0.57
**Angiotensin receptor blocker**	-	-	1.08 (0.97-1.21)	0.15
**Beta-blocker**	-	-	1.28 (1.15-1.42)	<0.001
**Statin**	-	-	0.89 (0.81-0.98)	0.02
**Thiazide diuretic**	-	-	1.11 (1.01-1.23)	0.03
**Calcium channel blocker**	-	-	1.24 (1.13-1.37)	<0.001
**Clopidogrel**	-	-	1.08 (0.88-1.33)	0.47
**Loop diuretic**	-	-	1.24 (1.06-1.45)	0.007
**Mineralocorticoid receptor antagonist**	-	-	1.00 (0.75-1.33)	0.99
**Digoxin**	-	-	2.54 (1.83-3.51)	<0.001
**Days on aromatase inhibitor (time-varying)**	-	-	1.00 (1.00-1.00)	0.11
**Days on tamoxifen (time-varying)**	-	-	1.00 (1.00-1.00)	0.10

© 2019 Abdel-Qadir et al. *JAMA Network Open.*

eTable 3. Results From the Multivariable Cause-Specific Hazard Regression Model Assessing the Relationship of Anthracycline and Trastuzumab Exposure With Atrial Fibrillation After Adjusting for Other Characteristics and Exposures of Early-Stage Breast Cancer Patients With Documented Chemotherapy Regimens

	**Model in full cohort**		**Model in patients aged ≥66 years**	
**Parameter**	**Hazard ratio (95% CI)**	**p-value**	**Hazard ratio (95% CI)**	**p-value**
**Age (per year)**	1.07 (1.06-1.08)	<.001	1.04 (1.01-1.07)	0.005
**Year of cohort entry (relative to 2007)**				
**2008**	1.02 (0.77-1.35)	0.91	1.62 (1.01-2.60)	0.05
**2009**	1.06 (0.80-1.42)	0.69	1.21 (0.73-2.00)	0.47
**2010**	1.10 (0.82-1.47)	0.55	1.56 (0.94-2.58)	0.09
**2011**	0.95 (0.69-1.31)	0.74	0.99 (0.57-1.1)	0.97
**2012**	1.02 (0.73-1.42)	0.91	1.38 (0.79-2.40)	0.26
**2013**	0.81 (0.56-1.16)	0.25	1.06 (0.58-1.95)	0.85
**2014**	0.78 (0.53-1.14)	0.20	1.08 (0.57-2.06)	0.81
**2015**	0.74 (0.49-1.14)	0.17	1.15 (0.56-2.35)	0.70
**2016**	1.12 (0.71-1.77)	0.63	1.58 (0.72-3.45)	0.25
**Income quintile (relative to 1 [lowest])**				
**2**	0.99 (0.80-1.24)	0.96	1.40 (1.00-1.97)	0.05
**3**	0.86 (0.68-1.09)	0.21	1.10 (0.76-1.60)	0.61
**4**	0.85 (0.68-1.07)	0.17	0.99 (0.68-1.44)	0.96
**5 (highest)**	0.78 (0.62-0.99)	0.04	0.96 (0.66-1.39)	0.82
**Unknown**	1.13 (0.27-4.69)	0.87	<0.01	<.001
**Urban residence (relative to rural)**	1.13 (0.91-1.40)	0.27	1.13 (0.82-1.55)	0.45
**Ischemic heart disease**	1.43 (1.08-1.90)	0.01	1.08 (0.70-1.66)	0.74
**Heart failure**	3.22 (2.18-4.76)	<.001	3.15 (1.88-5.26)	<.001
**Diabetes**	1.11 (0.92-1.35)	0.29	1.18 (0.90-1.55)	0.24
**Hypertension**	1.45 (1.23-1.71)	<.001	1.11 (0.81-1.52)	0.53

© 2019 Abdel-Qadir et al. *JAMA Network Open.*

**Peripheral Vascular disease**	1.08 (0.58-2.02)	0.80	0.61 (0.26-1.46)	0.27
**Stroke**	0.32 (0.04-2.55)	0.28	<0.01	<.001
**Chronic obstructive pulmonary disease**	1.62 (1.20-2.18)	0.002	1.58 (1.07-2.32)	0.02
**Chronic kidney disease**	0.88 (0.51-1.50)	0.64	0.68 (0.31-1.49)	0.34
**Breast cancer stage (relative to Stage 1)**				
**Stage 2**	1.08 (0.88-1.31)	0.46	1.29 (0.93-1.78)	0.13
**Stage 3**	1.33 (1.05-1.69)	0.02	1.49 (1.02-2.18)	0.04
**Unknown stage data**	1.00 (0.59-1.71)	0.99	0.57 (0.18-1.78)	0.33
**Left-sided breast cancer**	1.03 (0.89-1.19)	0.69	1.02 (0.82-1.26)	0.89
**Mastectomy (relative to lumpectomy)**	0.89 (0.75-1.06)	0.19	0.86 (0.66-1.12)	0.27
**Radiation exposure (time-varying)**	0.48 (0.40-0.57)	<.001	0.53 (0.41-0.69)	<.001
**Chemotherapy regimen (relative to non-anthracycline, non-trastuzumab chemotherapy)**				
**Anthracycline exposure (time-varying)**	1.12 (0.92-1.38)	0.26	1.23 (0.92-1.64)	0.16
**Trastuzumab exposure (time-varying)**	0.98 (0.74-1.30)	0.90	1.02 (0.71-1.47)	0.93
**Sequential therapy (time-varying)**	1.02 (0.79-1.31)	0.89	1.04 (0.71-1.55)	0.83
**Prescription medications (data only available for patients aged ≥ 66 years)**				
**ACE inhibitor**	-	-	1.02 (0.76-1.37)	0.90
**Angiotensin recepeptor blocker**	-	-	1.09 (0.81-1.47)	0.56
**Beta-blocker**	-	-	1.28 (0.96-1.71)	0.10
**Statin**	-	-	0.82 (0.64-1.06)	0.13
**Thiazide diuretic**	-	-	1.27 (0.97-1.65)	0.08
**Calcium channel blocker**	-	-	1.26 (0.96-1.64)	0.09
**Clopidogrel**	-	-	1.53 (0.82-2.86)	0.18
**Loop diuretic**	-	-	1.24 (0.74-2.07)	0.42
**Mineralocorticoid receptor antagonist**	-	-	0.80 (0.33-1.91)	0.61
**Digoxin**	-	-	4.76 (2.09-10.84)	<0.001
**Days on aromatase inhibitor (time-varying)**	-	-	1.00 (1.00-1.00)	0.97
**Days on tamoxifen (time-varying)**	-	-	1.00 (1.00-1.00)	0.50

© 2019 Abdel-Qadir et al. *JAMA Network Open.*
